# Alar cartilage—an alternative for spreader graft in primary rhinoplasty

**DOI:** 10.1007/s00238-017-1336-5

**Published:** 2017-07-12

**Authors:** Carlos Oscar Uebel, Renato Matta

**Affiliations:** Division of Plasic Surgery –PUCRS, Porto Alegre, Brazil

**Keywords:** Primary rhinoplasty, Inverted “V” deformity, Internal valve deficiency, Deviated nose, Spreader graft, Alar cartilage

## Abstract

**Background:**

Alar cartilage can be very useful for tip and dorsum grafts. Depending on its size and thickness, it can be an important alternative for spreader grafts to improve endonasal functional deficiencies, correct deviated noses, and prevent inverted “V” deformities. Caucasian patients with bulbous tips are the most common candidates to achieve such benefits. It is easy to obtain and to frame into a desired graft.

**Methods:**

The authors describe a surgical technique using the alar cartilages as spreader grafts. All Caucasian patients with bulbous tips who underwent primary rhinoplasty were included. All patients have been evaluated after 3 to 4 months and after 1 and 2 years by aesthetical and functional criteria.

**Results:**

Thirty-four patients (28 female and 6 male) underwent this procedure between 2001 and 2015: 94% reported a better airflow, 91% reported very good aesthetic results and were very satisfied 2 years postoperatively, and 12% had nasal deviations that were corrected with a one side double-layered spreader grafts. Two patients presented supra-tip deformities and one patient had a columella scar that was revised surgically. No cases of inverted “V” deformity were reported 2 years postoperatively.

**Conclusions:**

Patients with functional satisfaction and with a straight and smooth dorsum seem to be the most important benefits that were achieved with this technique using alar cartilage spreader grafts, an alternative that can be offered to improve airflow and to prevent deviated and inverted “V” deformities.

Level of Evidence: IV, therapeutic study.

## Introduction

Rhinoplasty techniques date back to the nineteenth century, as first described by Roe [1]. Thereafter, Joseph [2] devised an endonasal technique that is still performed by many surgeons today. Rethi described the open transcolumellar approach in 1929 [3], and more recently, Vogt [4] brought further advances to the open rhinoplasty approach.

One of the greatest challenges in rhinoplasty is maintaining endonasal integrity and functional nasal vault activity [5, 6]. The endonasal vaults are the narrowest part of the airway. These structures are defined by the junction between the septum and the caudal superior lateral cartilages, forming an angle of 10–15° in Caucasians (leptorrhine) and a larger angle in people of African and Asian heritage (platyrrhine) [5, 7, 8].

Some issues can affect the complex junction between the alar and superior lateral cartilages stretching between the lateral nasal wall and the terminal part of the inferior turbinate [1, 9]. Reasons for obstruction can be congenital, traumatic, or iatrogenic, particularly after a rhinoplasty [10]. Large resections of alar cartilage from the lateral crus, dorsal hump removal, and lateral osteotomies can reduce physiologic support for the superior lateral cartilage, resulting in deviated structures [9, 11]. A resection of only 2 mm of vault cartilage is sufficient to flatten it [12]. Because of this, when a dorsum removal is performed, it is sometimes necessary to spread and reconstruct the medial vault to prevent a vault deficiency and an inverted “V” deformity.

Many authors have described different methods to improve nasal vault function, including spreader grafts [10], spreader flaps [13, 14], also known as turnover flaps [15], alar batten flaps [16], splay grafts [17], flaring sutures [18], the auto-spreading spring flap [6], or double-layered stepped spreader graft [19]. Overall, 81% of patients receiving spreader grafts experience significant nasal airflow improvement [20].

The authors present an alternative for spreader grafts obtained from the cephalic portion of the alar cartilage, especially for patients with alar cartilage hypertrophy, such as Caucasian and Middle Eastern patients. This technique can be useful to correct those deformities preserving intact material from the septum, chondrocostal, or auricular cartilage, diminishing the surgical time and morbidity.

## Methods

**Fig. 1:**
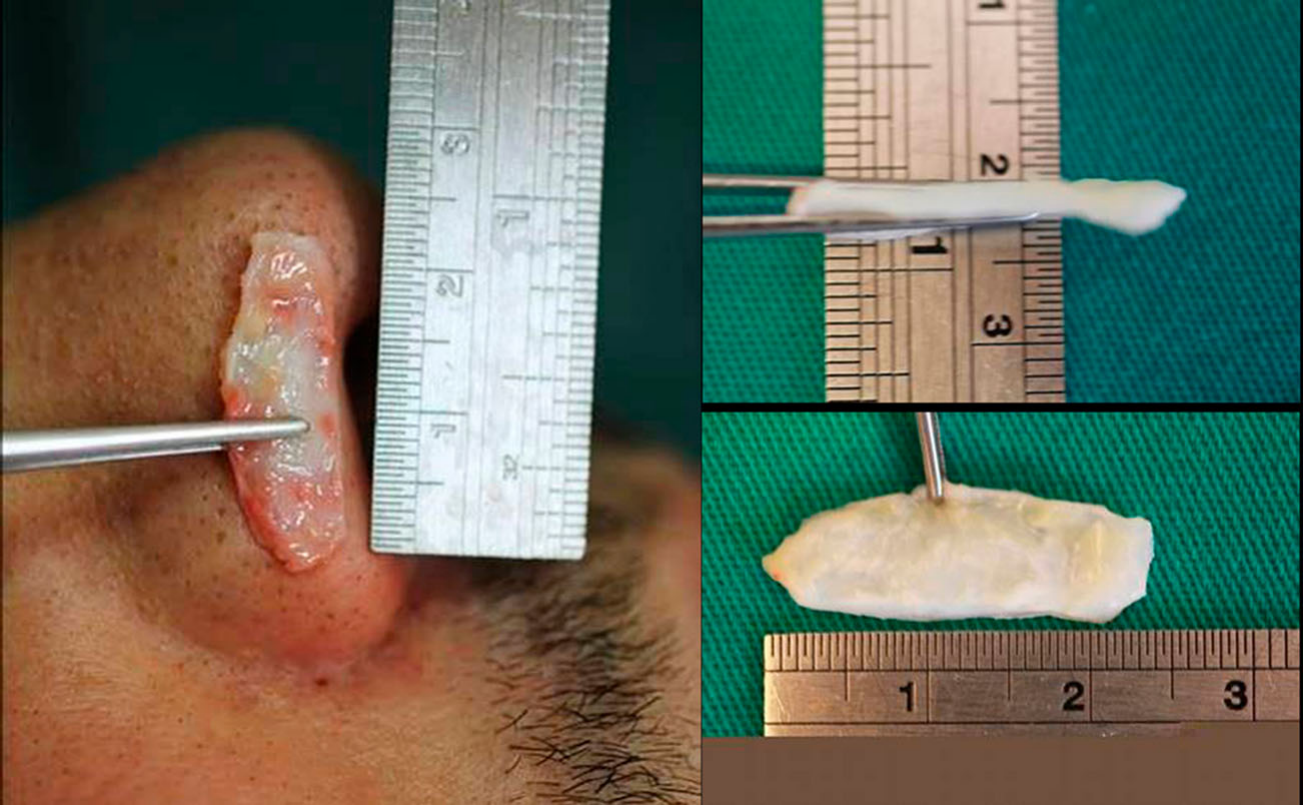
Alar grafts from our Caucasian patient series

**Fig. 2:**
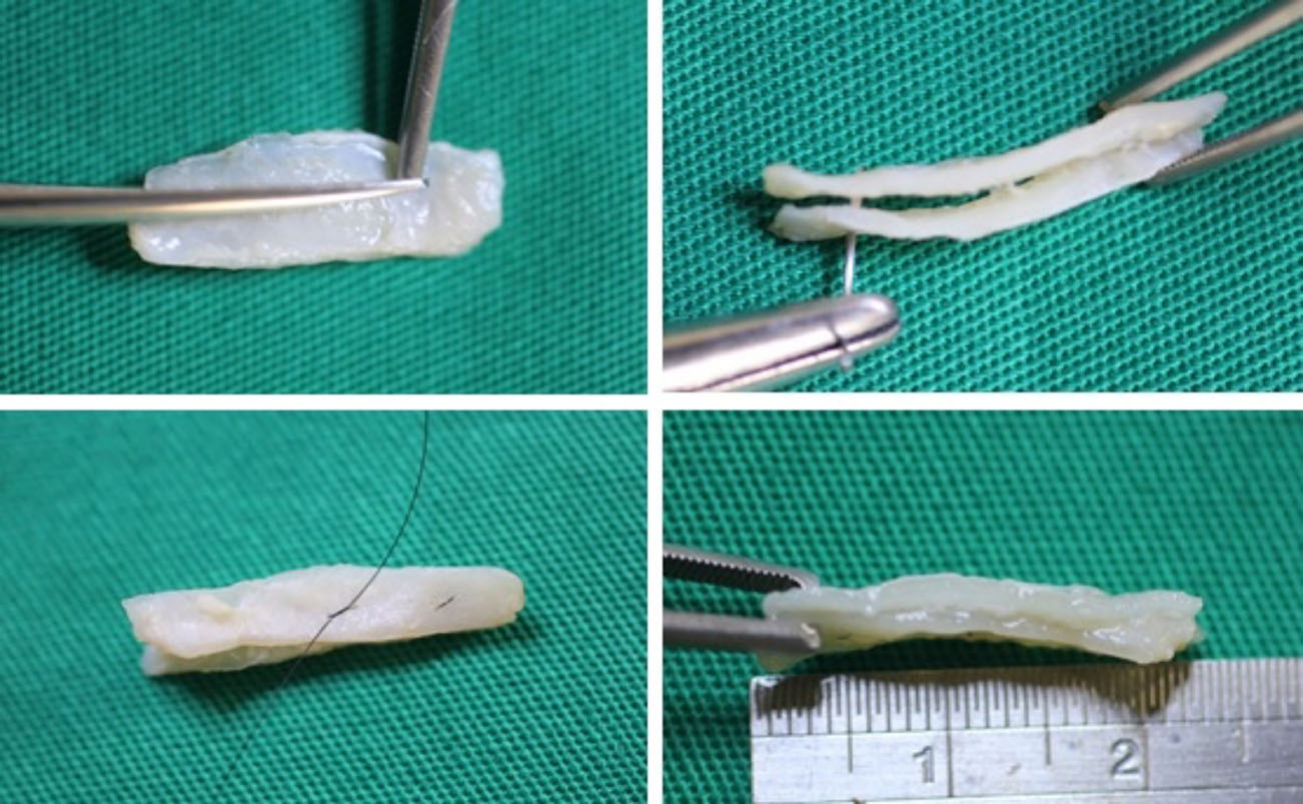
A double-layered alar spreader graft being prepared

Patients with pronounced and bulbous nasal tips were selected as good candidates to improve airflow and to prevent an inverted V deformity, from 2001 to 2015 in our Division of Plastic Surgery using alar cartilages as spreader grafts. In our study, we have considered patients with alar cartilages from 19 to 26 mm length, 9 to 12 mm width, and 2.5 to 3.5 mm thickness (Fig. 1). Four patients with nasal septal deviation were included, and in these cases, it was used a double-layered alar spreader graft (Fig. 2). There were excluded patients with previous rhinoplasty and with weak and smaller alar cartilages. They were evaluated monthly during fourth months and after 1 and 2 years postoperatively.

The main author performed the functional evaluation by direct inspection of the internal vault with speculum and asking the patient to perform the forced inspiration/expiration maneuver. The patients answered a questionnaire about the nasal airflow at the second year postoperative in a following scale: 1, better; 2, same before surgery; 3, worst on one side; and 4, worst on both sides. The aesthetic result was evaluated from a questionnaire by the patients as follows: 1, very good and very satisfied; 2, good and satisfied; 3, regular; and 4, bad. Besides this, an accurate study was done with pre- and postoperative pictures.

### Surgical technique

**Fig. 3:**
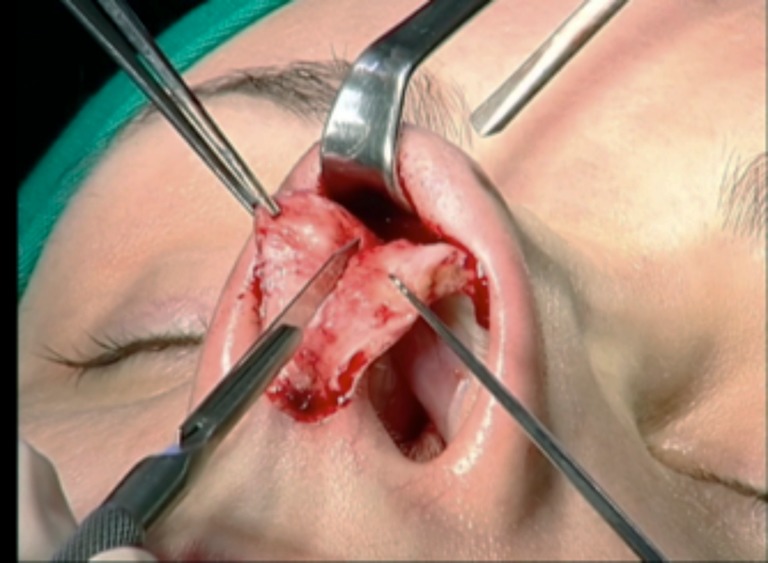
Septum exposure via sub-mucoperichondrial plane

Patients were placed under general anesthesia with 2,6-diisopropylphenol (Profolen® 10 mg/ml, Blau Farmacêutica S.A., SP, Brazil) or local in stand-by with midazolam (Dormonid® 15 mg/3 ml, Roche, RJ, Brazil) divided into 3 mg/2 cm^3^ portions delivered intravenously at approximately 30-min intervals or then propofol (10 mg/ml). Local infiltration was performed 15 min before incision with bupivacaine without epinephrine (Marcain® 0.25%, AstraZeneca, SP, Brazil) with epinephrine 1:120,000. An open approach was performed with an inferior “V” columellar incision extending to the nostril rim and subcutaneously to the glabella, with exposure of the alar cartilages and osteocartilaginous dorsum. The membranous septum was opened with septal exposure via the sub-mucoperichondrial plane (Fig. 3). The upper lateral cartilages were released from the septum by a submucosal approach, and the portion of the osteocartilaginous dorsum was resected with angular scissors and finalized with a rasp or osteotome.

**Fig. 4:**
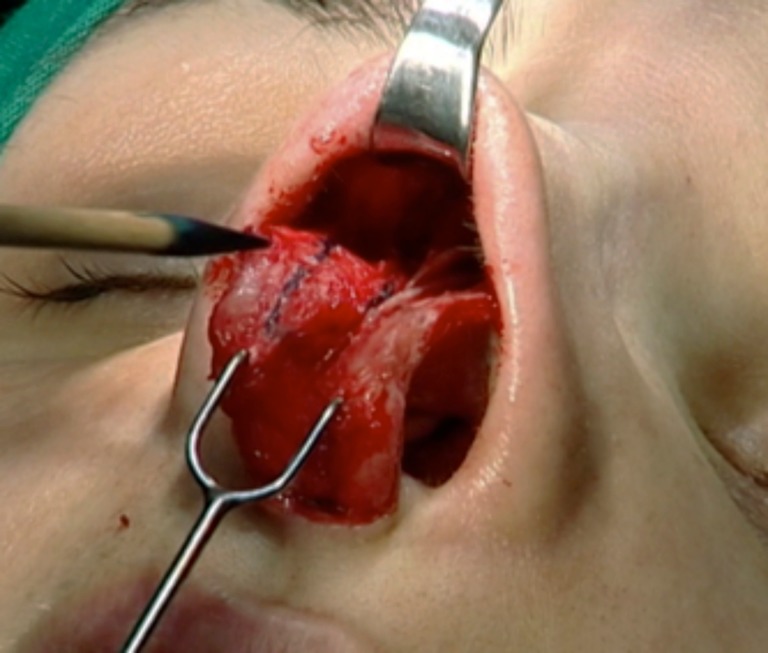
Marking the cephalic alar ellipse to be excised, leaving 3 to 4 mm of alar rim

**Fig. 5:**
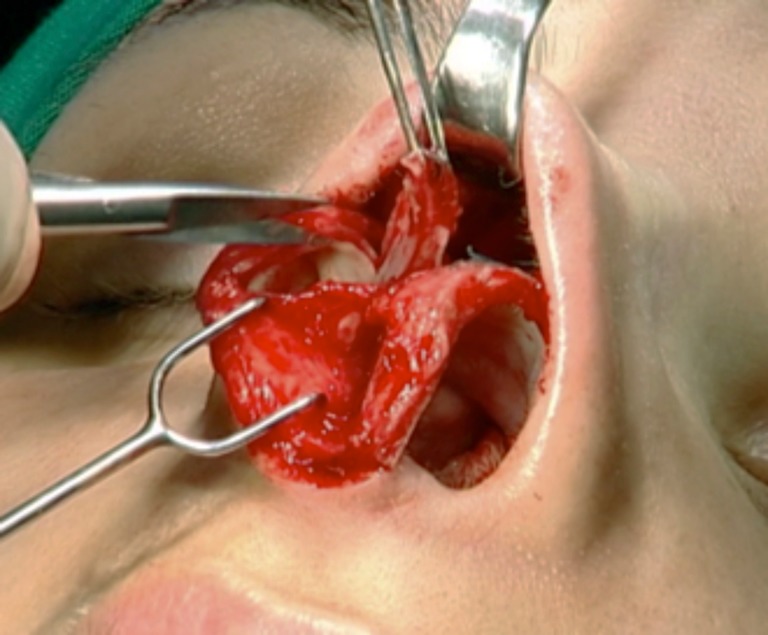
Alar resection “en bloc” with small nasal mucosa excess

**Fig. 6:**
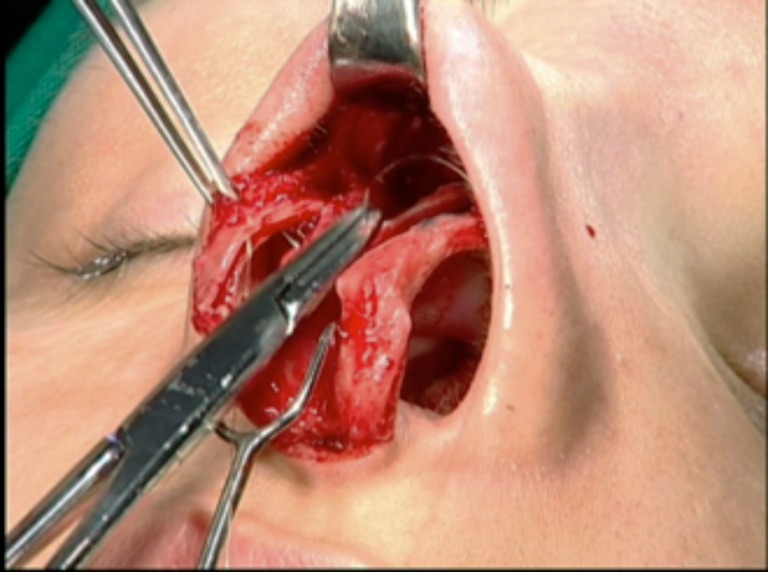
Suture of the opening structures, including mucosal layer

The next step was to identify and prepare the alar cartilage spreader grafts. A cephalic ellipse was removed with no less than 19 mm of length and 2 mm of thickness (Fig. 4). If the patient had a large bulbous tip, a resection “en bloc” including cartilage and a small inner portion of mucosa was performed (Fig. 5). Besides this, mucosal excision is performed mostly in patients to prevent ripples inside the external valve. The defect is closed with poliglecaprone 5–0 suture (Monocryl®, Ethicon, Johnson & Johnson do Brasil, SP, Brazil) (Fig. 6).

**Fig. 7:**
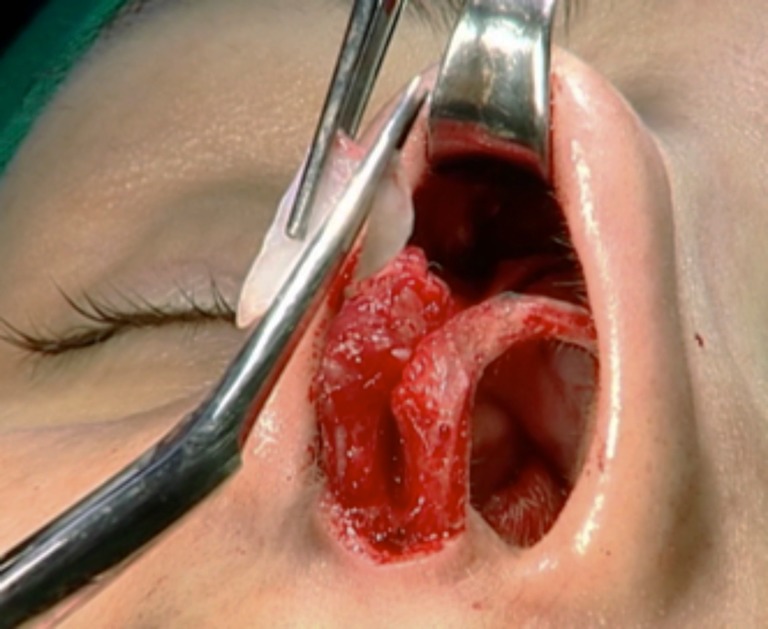
Trimming “alar cartilage spreader graft”

**Fig. 8:**
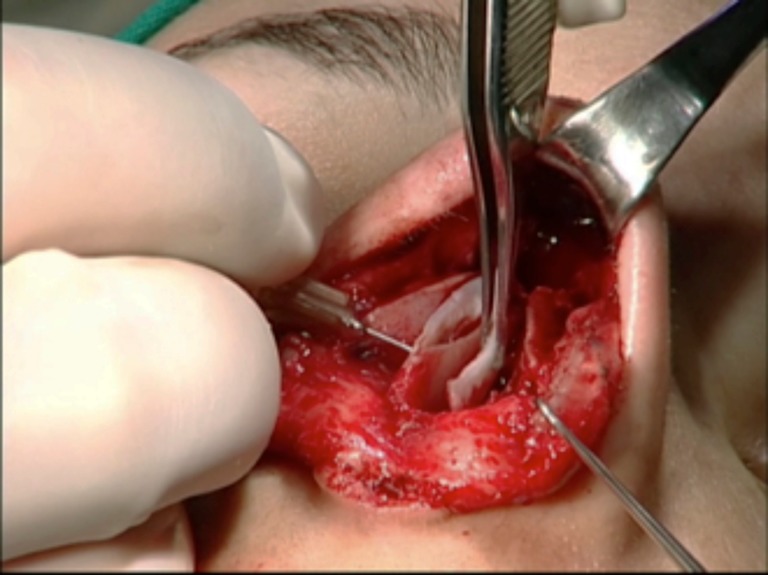
Both graft attachment with the convex side oriented medially. Note the thickness of the alar grafts in relation to the septal plate

**Fig. 9:**
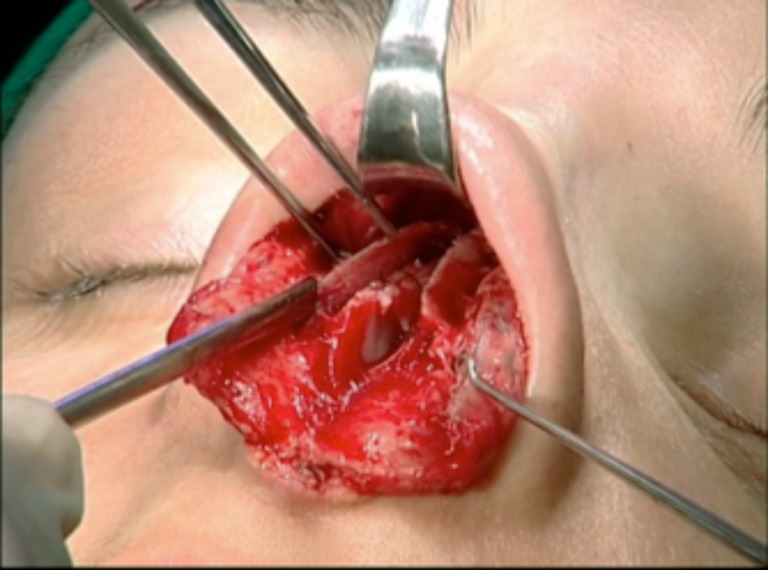
Trimming upper lateral cartilages. They can also be sutured over the dorsum

Both cartilages are trimmed, inserted into the superior septum as spreader grafts, and fixed with nylon 5–0 (Ethilon®, Ethicon, Johnson & Johnson do Brasil, SP, Brazil) (Figs. 7 and 8). If some excess of upper lateral cartilage is available, it can be excised or sutured over the dorsum covering the spreader grafts to produce a smooth and straight dorsum (Fig. 9). After closing the columellar and rim incisions with Monocryl 4–0, tape strips (Micropore®, 3M Health Care, St. Paul, MN, USA) are applied and an acrylic splint is fixed over the dorsum, where it will remain for 8 days. No endonasal packing was used.

## Results

**Fig. 10:**
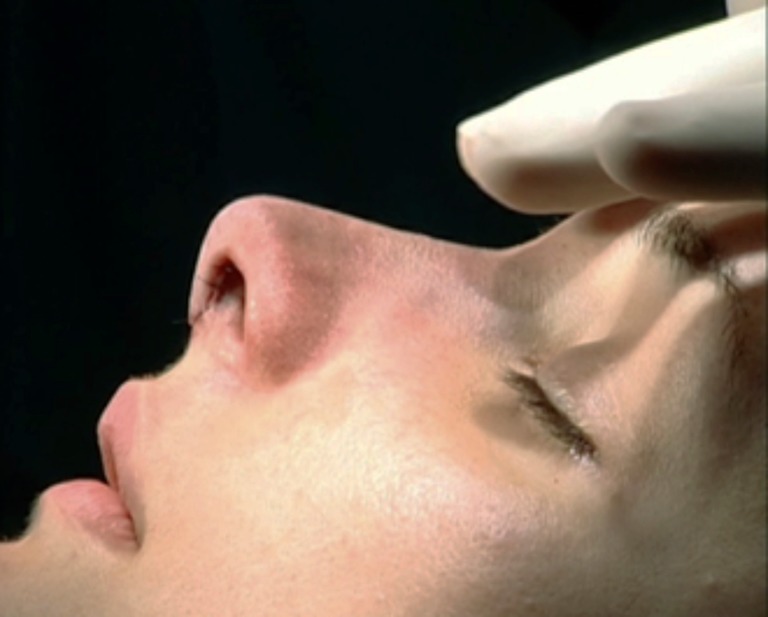
Dorsum immediate postoperative result

**Fig. 11:**
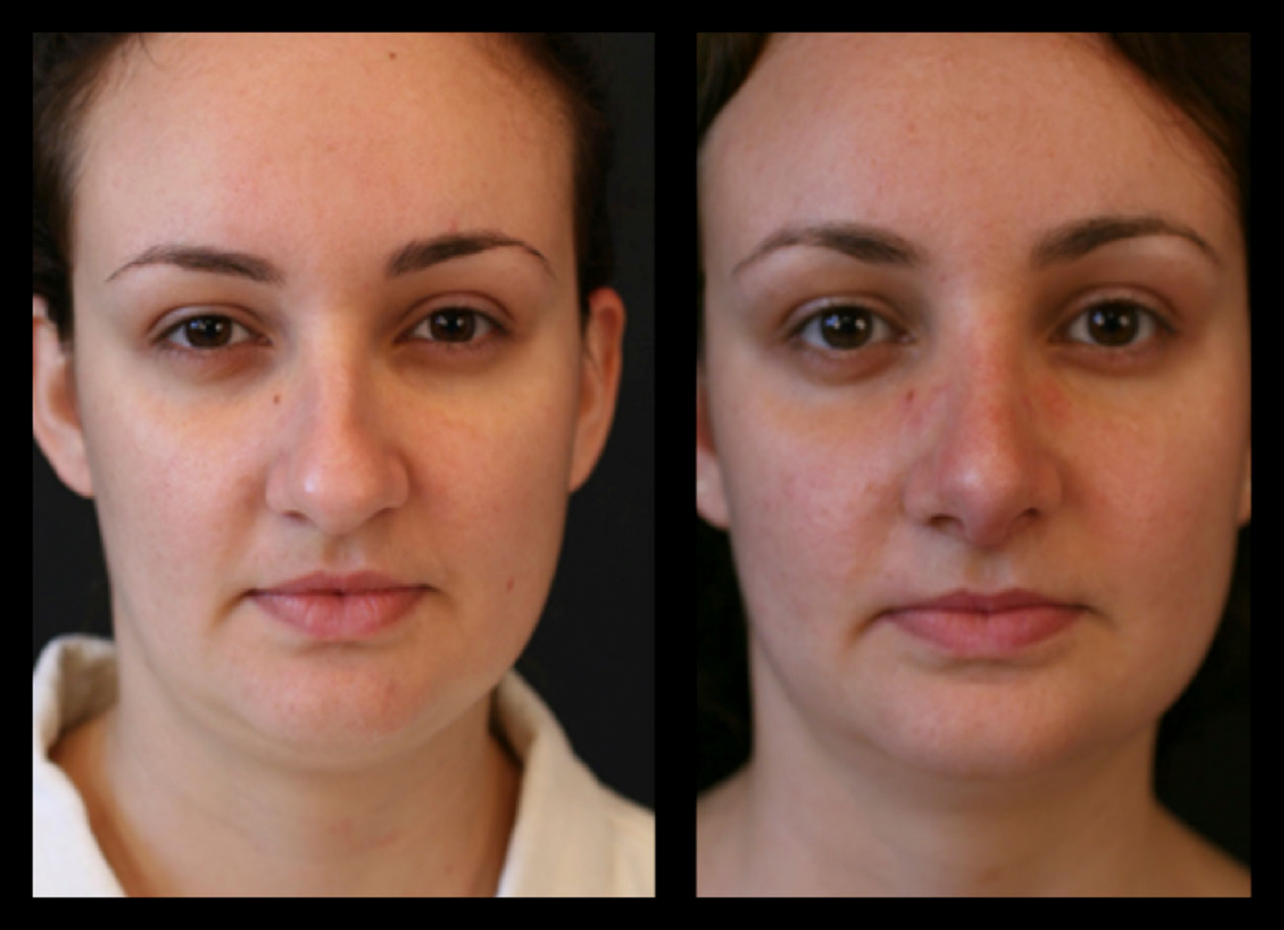
Thirty-two-year-old patient. Preoperative and 1-year postoperative result. Well-defined nasal dorsum, “eyebrow-nasal tip line,” and an optimal functional outcome

**Fig. 12:**
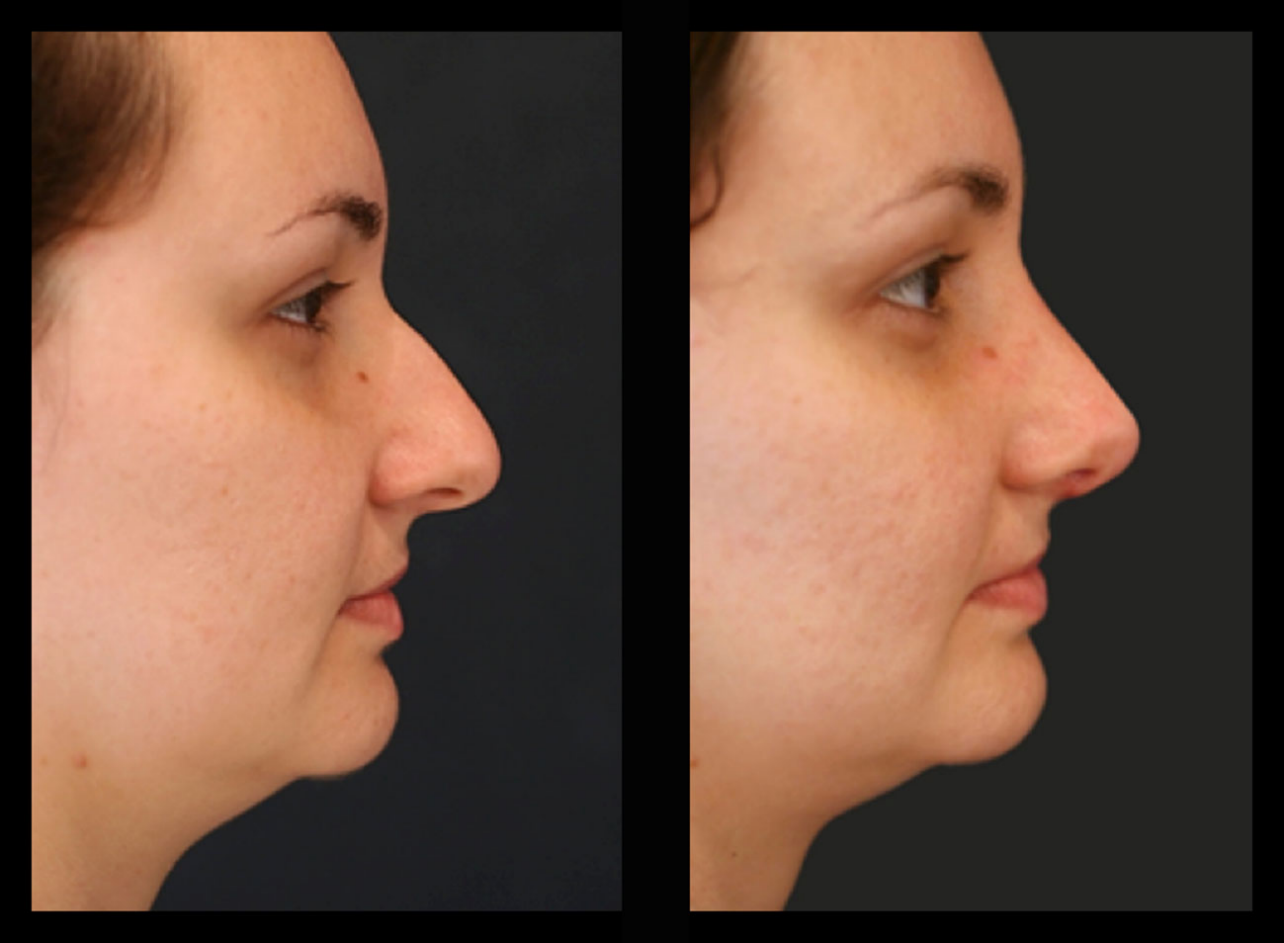
Same patient in lateral view

**Fig. 13:**
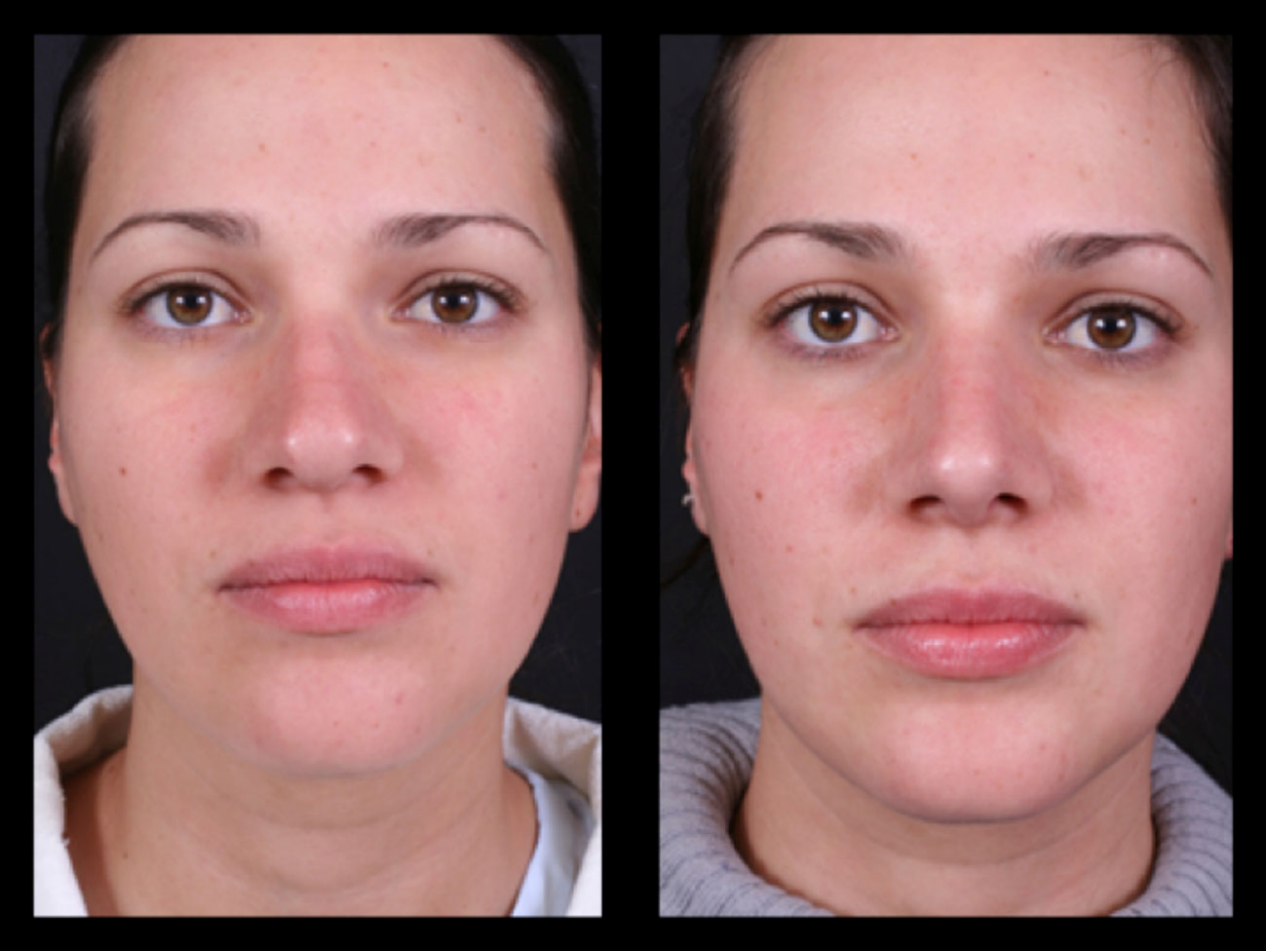
Twenty-six-year-old patient. Pre- and fourth month postoperative result. Straight dorsum lines and well-defined nasal tip

**Fig. 14:**
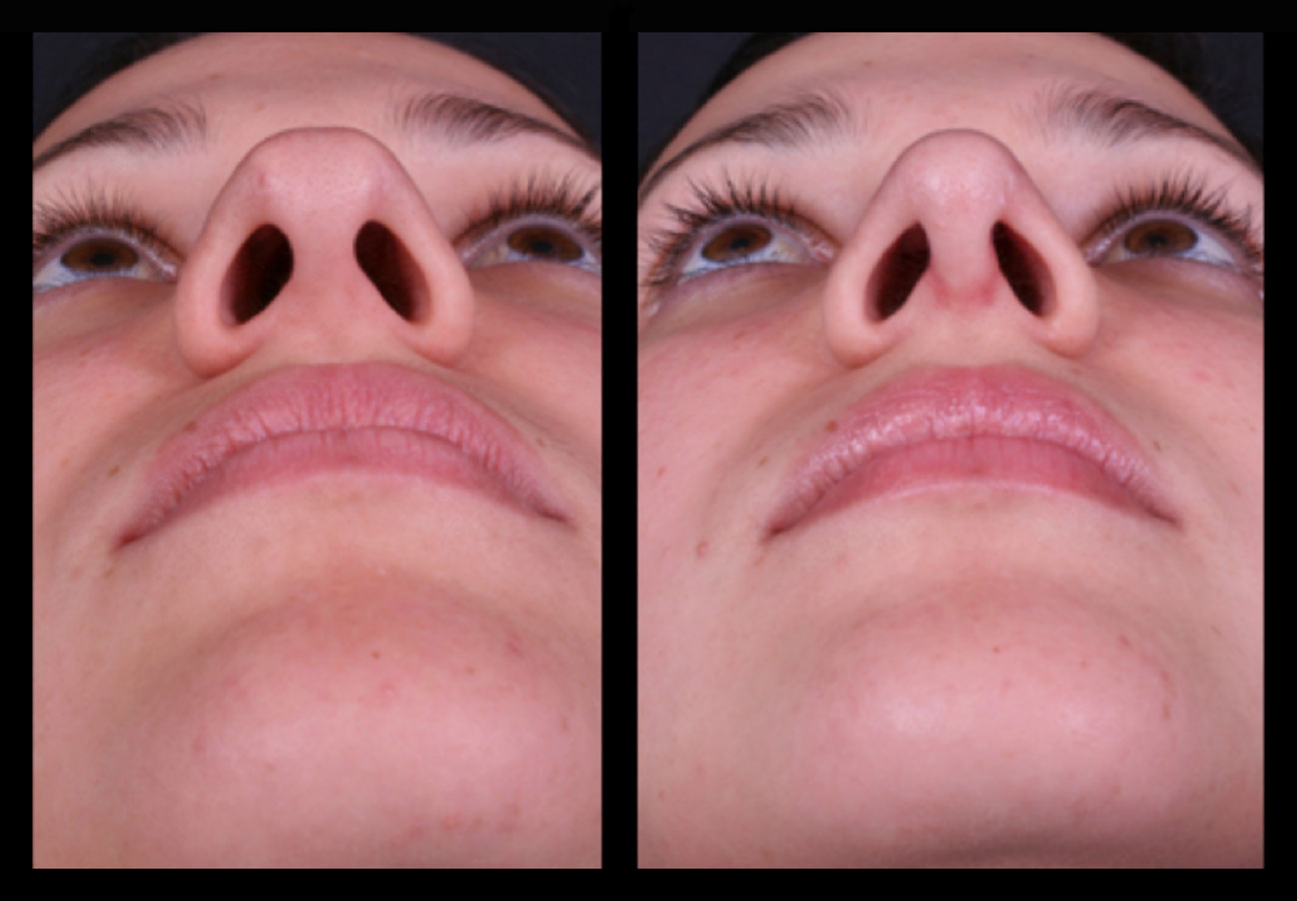
The same patient in inferior view

**Fig. 15:**
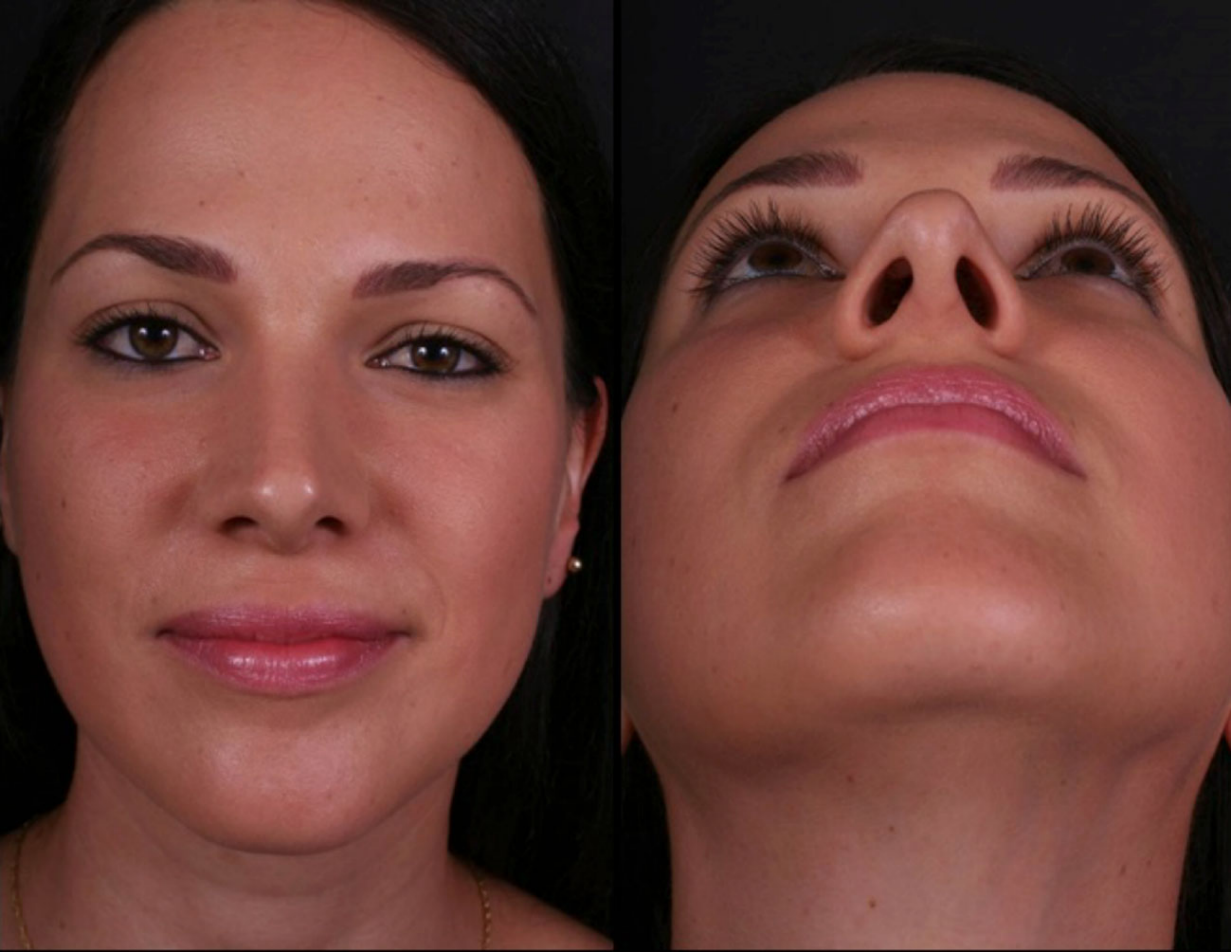
The same patient 5 years postoperatively

**Fig. 16:**
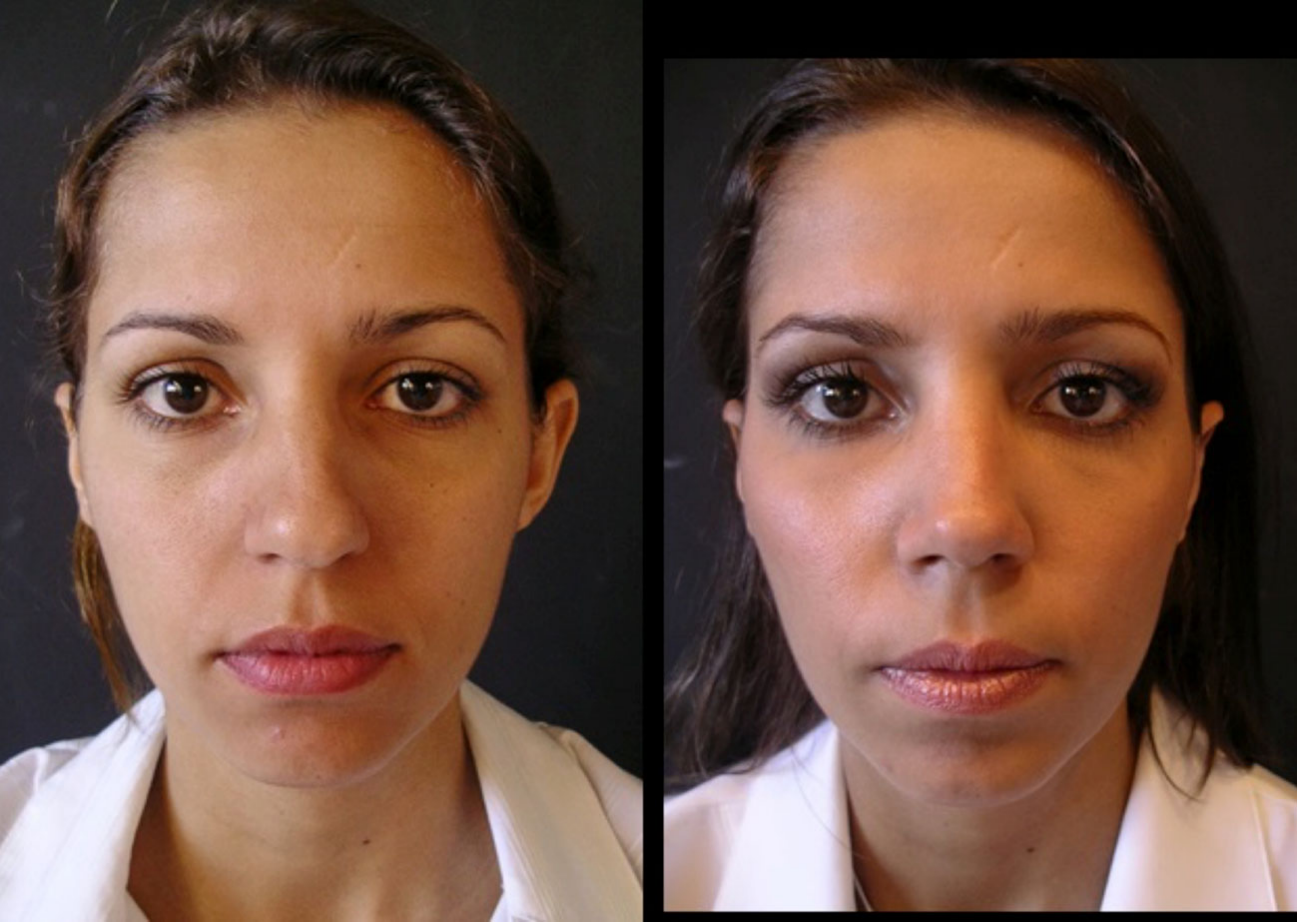
Twenty-six-year-old patient. Pre- and fourth month postoperative result. Straight dorsum lines and well-defined nasal tip

**Fig. 17:**
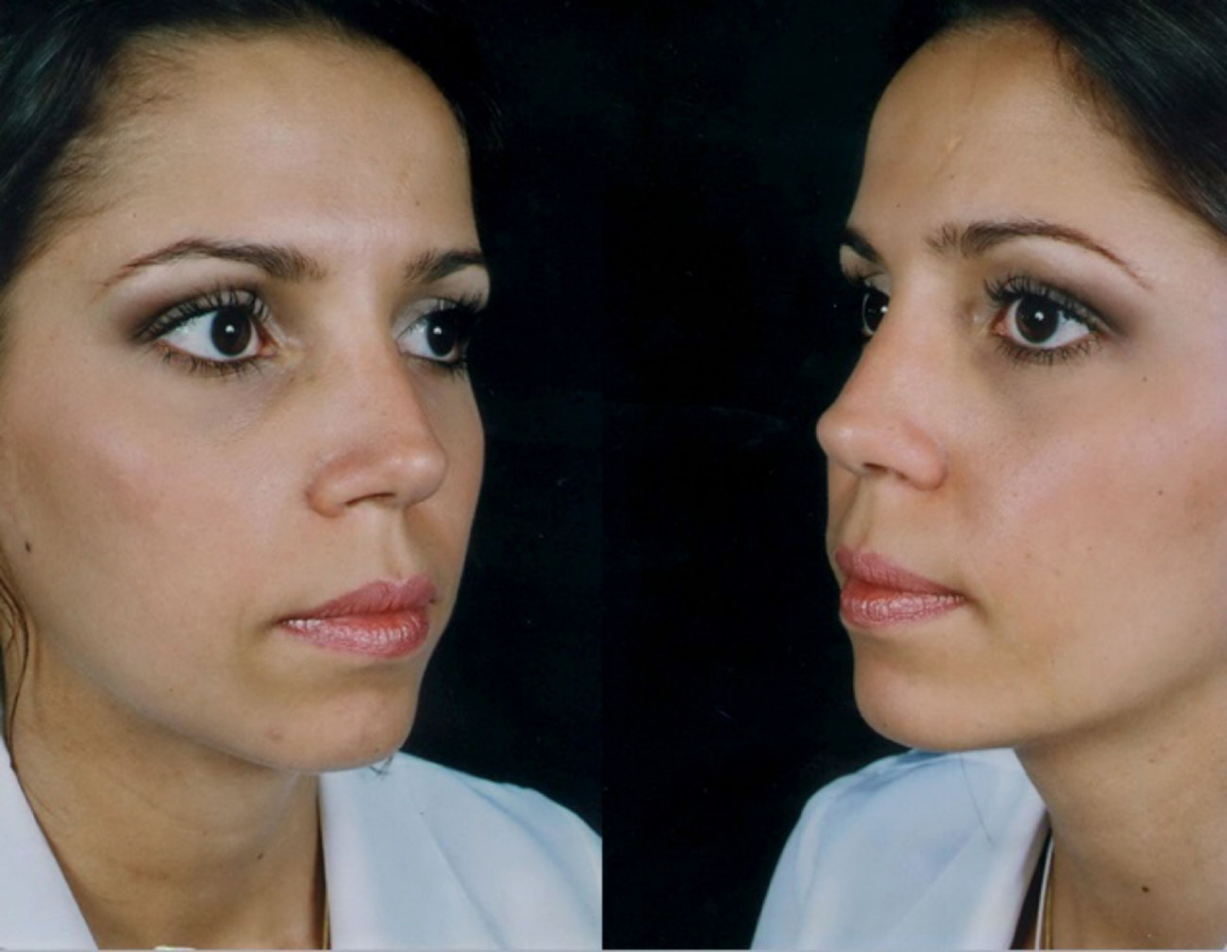
The same patient 5 years postoperatively in an oblique view

**Fig. 18:**
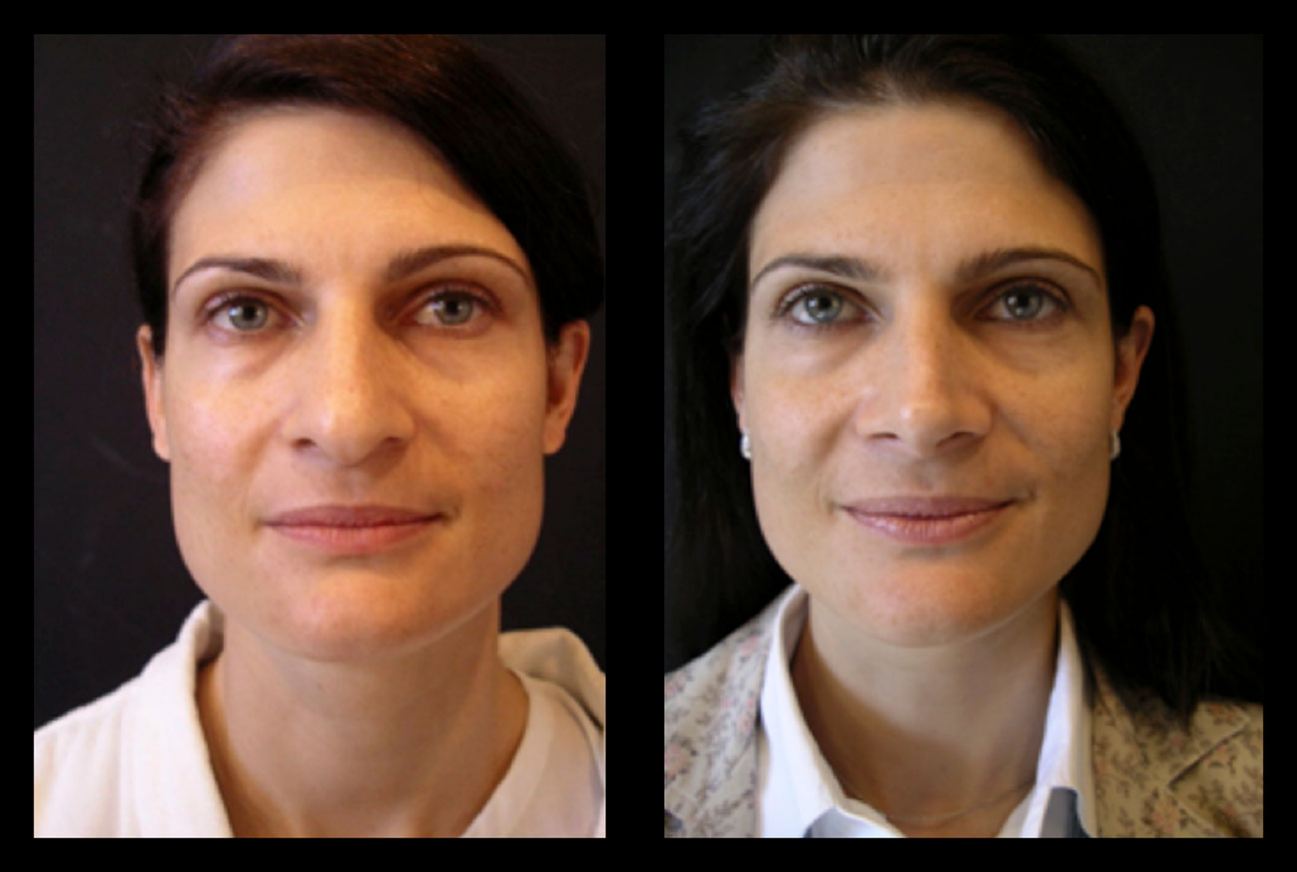
Thirty-six-year-old patient. Pre- and 2 years postoperative result

**Fig. 19:**
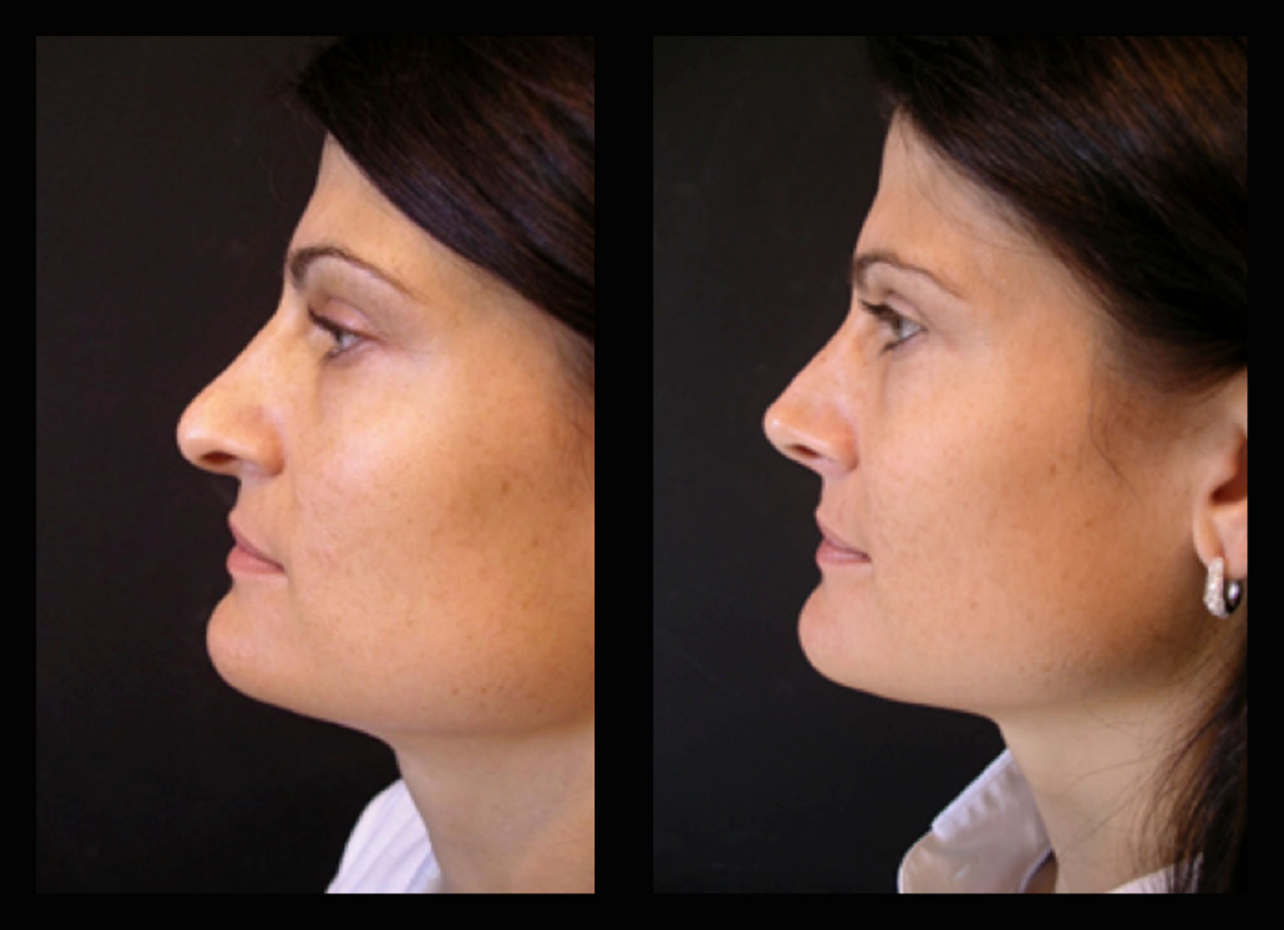
Same patient in lateral view

**Fig. 20:**
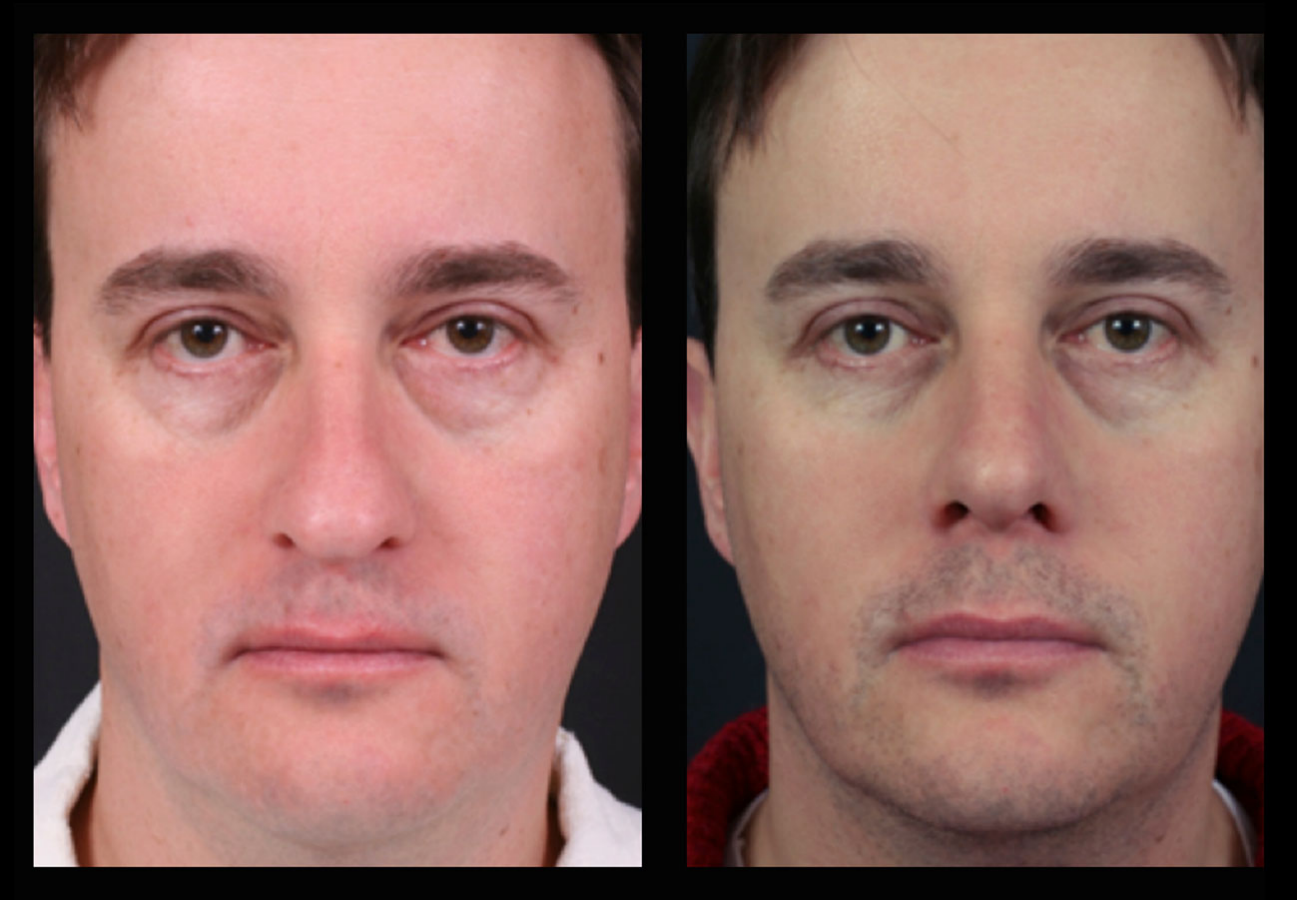
Forty-two-year-old male patient with a bulbous tip pre- and postoperative of 3 years

**Fig. 21:**
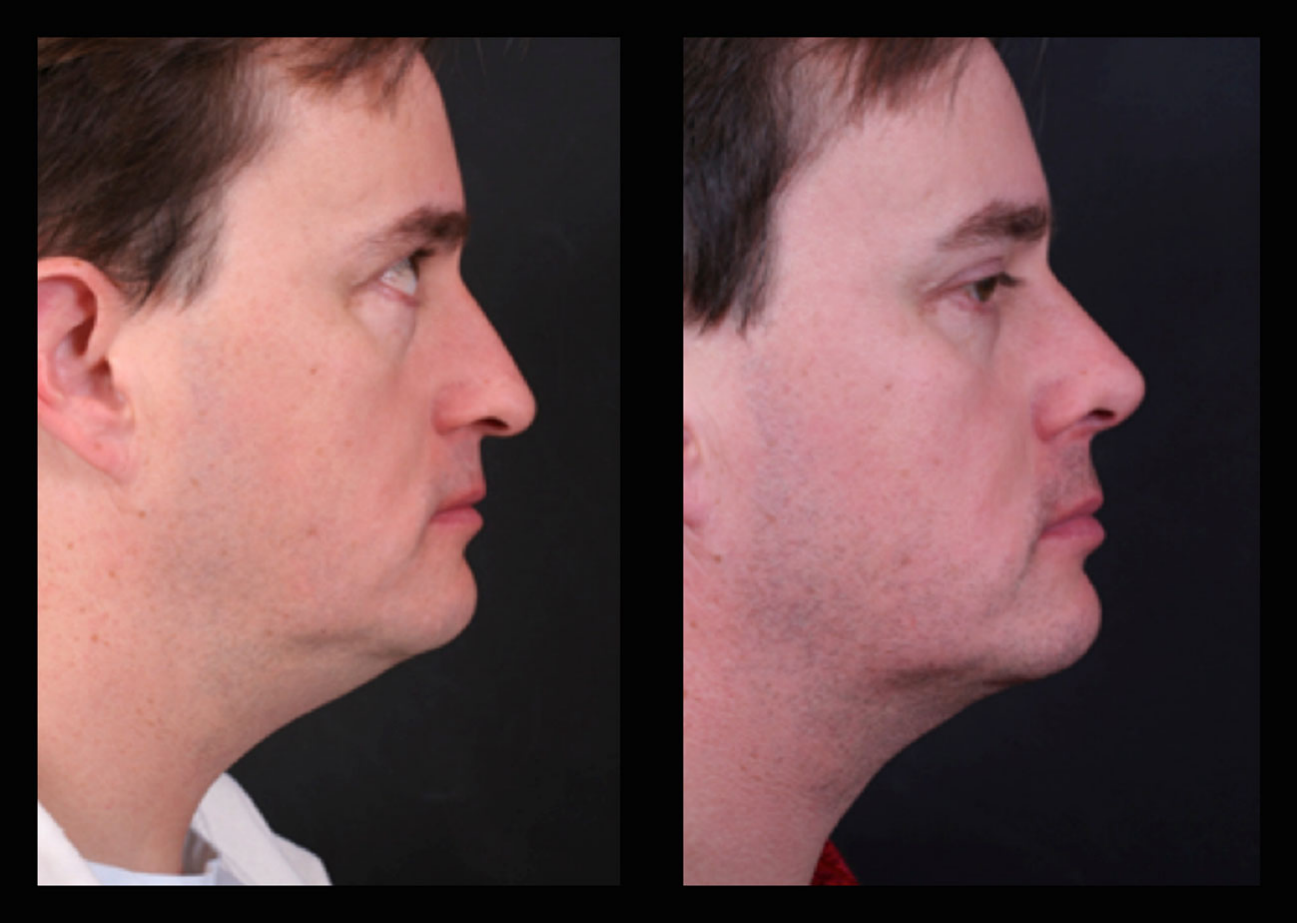
Lateral view of the same patient

Thirty-four patients (28 female and 6 male) underwent this procedure between 2001 and 2015 by an open rhinoplasty approach under general and local anesthesia; three of them stayed for one night at the hospital and the others went home in the same day of the surgery. The majority of them (94%) reported a better airflow, no patient reported worst functional result 1 year postoperatively, 6% reported worst airflow in one nasal side 2 years postoperatively, 91% reported a very good aesthetic results and were very satisfied 2 years postoperatively, and 12% had nasal deviations that were corrected with a one side double-layered spreader grafts. Two patients presented supra-tip deformities that were revised surgically and in one patient a columella scar revision. No cases of inverted “V” deformity were reported 2 years postoperatively. The patients’ features are summarized in Table 1. Representative cases are depicted in Figs. 10, 11, 12, 13, 14, 15, 16, 17, 18, 19, 20, and 21).

**Table 1:** Patients’ characteristics

Characteristic	No. of patients	Percentage
Sex (total)	34	100%
Female	28	82%
Male	6	18%
Age range	22–58	–
Follow-up of 2 years	34	100%
Follow-up of 5 years	23	68%
Lost to follow-up in 5 years	11	32%
Functional evaluation (second year postoperative)		
Better airflow	32	94%
Same before surgery	0	–
Worst in one side	2	6%
Worst in both sides	0	–
Aesthetic result (second year postoperative)		
Very good and very satisfied	31	91%
Good but it could be better	3	9%
Other	0	–
Patients with nasal septal deviation	4	12%
Surgical re-interventions	3	9%

## Discussion

The use of cartilage as spreader graft is inexpensive and practical should be the first choice when available [6]. The most common donor area is the septum because of its strong and straight cartilage. A chondrocostal graft offers the advantage of a large amount of donor material, allowing the surgeon to shape long, straight, and thick pieces. However, it has disadvantages of requiring sculpture to achieve an adequate form and the need to harvest from the sixth or seventh rib, with the possibility of postoperative morbidity. Auricular cartilage is easier to obtain, but the graft will not be straight and can curl over after implantation [21, 22]. Spreader flaps from the upper lateral cartilages can be another option for patients with a large humpectomy as described by Gruber et al. [13], Berkowitz [23], Oneal and Berkowitz [24], and Seyhan [25]. Alloplastic and cadaveric materials have been used in some patients [26], but at high cost, and with the possibility of infection and extrusion [6]. Calcium hydroxyapatite [27] and hyaluronic acid [28] have been described for use as spreader graft material. It has been used at the sub-mucoperichondrial level, as have high-density polyethylene (HDPP) plates [22].

Alar cartilages can be another option for spreader grafts depending on its structure and dimensions. In 1979, Zelnik and Gingrass [29] described average Caucasian alar cartilage dimensions of 21.1-mm length by 1.2-mm width. An anatomical study of Asians found an average length of 17.9 (±2.28) mm, width of 10.0 (±1.31) mm, and thickness of 0.54 (±0.09) mm in both genders [30]. In another study of Iranians, the reported average was 23.4 (±2.7) mm length, 10.8 (±1.29) mm width, and 1.0 (± 0.15) mm thickness [31]. More recently, Daniel et al. [32] described an average thickness of 0.5 mm at the lateral crura [32]. In our series of 34 Caucasian patients, we have observed alar cartilages from 19 to 26 mm of length, width of 9 to 12 mm, and from 2.5 to 3.5 mm of thickness.

Described by Sheen in 1984 [33], the spreader graft is considered a well-established technique to improve the internal vault. It not only improves the angle between septum and superior lateral cartilages but also improves the aesthetic appearance of the nasal dorsum “eyebrow-nasal tip lines” [5, 6, 34]. Initially, the spreader graft was described to correct the inverted “V” deformity caused by large osteocartilaginous dorsum removal [35]. It is also used for nasal roof reconstruction after dorsum removal and for reconstruction of asymmetric noses [34]. The most common challenge involves fixing the spreader grafts to maintain the symmetry of the nasal dorsum [36]. Some techniques have been described to help maintain these grafts, including the narrow dorsal tunnel, trans-cutaneous, and trans-septal sutures [34].

According to the Bernoulli principle [5], reduced pressure is transmitted to the lateral walls during inspiration, thereby narrowing the internal nasal vault. Air passing through the nasal vaults at high speed lowers intraluminal pressure, producing an inspiratory collapse [35]. This effect can be estimated by acoustic rhinometry [37, 38], one of few available tests to identify nasal airflow and nasal vault deficiencies [39].

Some details of this technique should be considered, such as choosing an adequate patient to perform the procedure. A strong, thick, and long lateral crura is necessary, especially in those large and bulbous tips. The alar cartilage slice should have a thickness like the septum from 2 to 3 mm and a length sufficient to spread the middle internal valve from the anterior septal angle to the osteocartilaginous junction from a minimum of 19 to 26 mm in length. The cartilage graft should be placed and adapted with the natural convex side oriented medially for better opening of the nasal valve angle. After placement of the “alar cartilage spreader graft,” any excess ULC can be trimmed or sutured over the dorsum, covering the spreader grafts, to create a smooth and straight dorsum, as described by Cerkes [40] and others [28, 32]. Another issue, mostly found in large bulbous nose, is the redundant inner nasal mucosa that can be excised “en bloc” and removed from the alar cartilage. Although we have a mucosa retraction, this can improve and bring up the nasal tip and prevent ripples inside the nasal valve. In cases we need a stronger and more structured spreader graft, especially in nasal deviation, a double-layered alar graft can be used (Fig. 2).

This is a very simple, brief, and predictable procedure that has an excellent teaching curve. After 1 year, postoperative patients have had acceptable functional result, and after 2 years, a good aesthetic results. Further studies and conclusions should be done in the future with a larger series of patients.

## Conclusions

The alar cartilage spreader graft, in selected patients, is an alternative and effective procedure to improve dorsum deformities, to prevent inverted “V” deformities, and to improve airway flow in the internal nasal valve deformities. It is a predictable technique with a low complication rate with a simple and short surgical protocol.
